# Common germline variants within the *CDKN2A/2B* region affect risk of pancreatic neuroendocrine tumors

**DOI:** 10.1038/srep39565

**Published:** 2016-12-23

**Authors:** Daniele Campa, Gabriele Capurso, Manuela Pastore, Renata Talar-Wojnarowska, Anna Caterina Milanetto, Luca Landoni, Evaristo Maiello, Rita T. Lawlor, Ewa Malecka-Panas, Niccola Funel, Maria Gazouli, Antonio De Bonis, Harald Klüter, Maria Rinzivillo, Gianfranco Delle Fave, Thilo Hackert, Stefano Landi, Peter Bugert, Franco Bambi, Livia Archibugi, Aldo Scarpa, Verena Katzke, Christos Dervenis, Valbona Liço, Sara Furlanello, Oliver Strobel, Francesca Tavano, Daniela Basso, Rudolf Kaaks, Claudio Pasquali, Manuel Gentiluomo, Cosmeri Rizzato, Federico Canzian

**Affiliations:** 1 Department of Biology, University of Pisa, Pisa, Italy; 2 Digestive and Liver Disease Unit, S. Andrea Hospital, ‘Sapienza’ University of Rome, Rome, Italy; 3 Genomic Epidemiology Group, German Cancer Research Center (DKFZ), Heidelberg, Germany; 4 Dept of Digestive Tract Diseases, Medical University of Lodz, Poland; 5 Department of Surgery, Oncology and Gastroenterology (DISCOG), Pancreatic and Digestive Endocrine Surgery, University of Padova, Padova, Italy; 6 Department of Surgery, University and Hospital Trust of Verona, Verona, Italy; 7 Department of Oncology, IRCCS Scientific Institute and Regional General Hospital “Casa Sollievo della Sofferenza”, San Giovanni Rotondo, Italy; 8 ARC-NET: Centre for Applied Research on Cancer, University and Hospital Trust of Verona, Verona, Italy; 9 Department of Translational Research and New Technologies in Medicine and Surgery, University of Pisa, Pisa, Italy; 10 Department of Basic Medical Sciences, Laboratory of Biology, Medical School National and Kapodistrian University of Athens, Greece; 11 Department of Surgery, IRCCS Scientific Institute and Regional General Hospital “Casa Sollievo della Sofferenza”, San Giovanni Rotondo, Italy; 12 Mannheim Institute of Transfusion Medicine and Immunology, Heidelberg University, Medical Faculty Mannheim, German Red Cross Blood Service Baden-Württemberg – Hessen, Mannheim, Germany; 13 Department of General Surgery, University Hospital Heidelberg, Heidelberg, Germany; 14 Blood Transfusion Service, Azienda Ospedaliero-Universitaria Meyer, Florence, Italy; 15 Division of Cancer Epidemiology, German Cancer Research Center (DKFZ), Heidelberg, Germany; 16 Department of Surgery, Konstantopouleion General Hospital Nea Ionia, Greece; 17 Department of Medicine (DIMED), Laboratory Medicine, University of Padova, Padova, Italy; 18 Division of Gastroenterology and Research Laboratory, IRCCS Scientific Institute and Regional General Hospital “Casa Sollievo della Sofferenza”, San Giovanni Rotondo, Italy

## Abstract

Pancreatic neuroendocrine tumors (PNETs) are heterogeneous neoplasms which represent only 2% of all pancreatic neoplasms by incidence, but 10% by prevalence. Genetic risk factors could have an important role in the disease aetiology, however only a small number of case control studies have been performed yet. To further our knowledge, we genotyped 13 SNPs belonging to the pleiotropic *CDKN2A/B* gene region in 320 PNET cases and 4436 controls, the largest study on the disease so far. We observed a statistically significant association between the homozygotes for the minor allele of the rs2518719 SNP and an increased risk of developing PNET (OR_hom_ = 2.08, 95% CI 1.05–4.11, p = 0.035). This SNP is in linkage disequilibrium with another polymorphic variant associated with increased risk of several cancer types. In silico analysis suggested that the SNP could alter the sequence recognized by the Neuron-Restrictive Silencer Factor (NRSF), whose deregulation has been associated with the development of several tumors. The mechanistic link between the allele and the disease has not been completely clarified yet but the epidemiologic evidences that link the DNA region to increased cancer risk are convincing. In conclusion, our results suggest rs2518719 as a pleiotropic *CDKN2A* variant associated with the risk of developing PNETs.

Pancreatic neuroendocrine tumors (PNETs) are heterogeneous neoplasms which are generally considered rare with an incidence around 0.40 per 100,000 subjects/year^1^,^2^ but their incidence has more than doubled in the last 30 years^1^,^3^,^4^. PNETs represent only 2% of all pancreatic neoplasms by incidence, but 10% by prevalence^2^,^5^. PNETs have been poorly studied, due to their rarity, and compared to other cancer types very little is known regarding either environmental or genetic risk factors for their occurrence. The role of traditional cancer risk factors such as smoking and alcohol consumption seems controversial^6^,^7^,^8^. On the contrary, type two diabetes (T2D) and family history of cancer have been consistently associated with PNET risk^7^,^8^.

Therefore genetic risk factors may have a role in disease aetiology, at least in a subset of PNET cases. Despite this, the impact of the genetic variability in the disease incidence is poorly understood. Only a small number of case-control studies have been performed to uncover the genetic susceptibility to PNETs^9^,^10^,^11^,^12^ and no genome wide association study (GWAS) has been performed yet.

With the goal of further our knowledge on PNET susceptibility we have performed a case-control study considering the genetic variability of the *CDKN2A/2B* region. The selection of the region was motivated by a fairly large amount of evidences pointing to a key role of this locus in pancreatic cancer onset and prognosis. For example *CDKN2A* is commonly mutated or de-regulated in both endocrine and exocrine pancreatic cancer^13^. Genetic polymorphisms in the locus have been reported to be associated with type two diabetes mellitus (T2DM), which is a one of the few suggested risk factors for PNETs^14^,^15^,^16^, suggesting a shared genetic background between T2DM and PNETs. Genetic variants belonging to the *CDKN2A/2B* region have been identified through GWAS as susceptibility markers for several human traits and diseases, including a large number of tumor types^17^,^18^,^19^,^20^,^21^,^22^,^23^,^24^. In addition we have recently showed the association of the *CDKN2A/2B*-rs3217992 SNP with increased risk of pancreatic ductal adenoma carcinoma (PDAC)^25^. The pleiotropic role of this region is justified by its crucial role in the regulation of the cell cycle^26^,^27^. Finally in a manuscript investigating the genetic susceptibility to endocrine tumors (NETs) Ter-Minassian and colleagues have suggested the association of four SNPs (representing two independent signals because of high linkage disequilibrium (LD)) in this region and an increased risk of the disease^11^.

Our hypothesis was that common genetic variability at the locus could modulate the risk of developing PNETs, as it has been shown for other cancer types.

## Results

### Data filtering and quality control

The origin of the population by country is shown in Table 1. None of the SNPs were out of Hardy-Weinberg equilibrium (HWE) in controls (p > 0.05). A total of 311 subjects (17 PNET cases and 294 controls) were removed after genotyping because they had a call rate < 75%. After the removal of these subjects the average SNP call rate was 95.54% with a minimum of 89.79% (rs3731246) and a maximum of 99.15% (rs3218009). The quality control analysis showed a concordance rate of 99.68% between the duplicate samples. After exclusions, 320 cases and 4,436 controls were used for statistical analyses.

### SNPs main effect

We observed a statistically significant association between the carriers of the A allele of the rs2518719 SNP and an increased risk of developing PNET (OR_hom_ = 2.08, 95% CI 1.05–4.11, p = 0.035). The association was statistically significant only comparing the rare and common homozygous individuals. None of the other SNPs showed any statistically significant associations. The frequencies and distributions of the genotypes, the odds ratios (ORs) for the association of each polymorphism with PNET risk and relative confidence intervals (CI) are shown in Table 2.

### Possible functional effects

We used several bioinformatic tools to predict possible functional relevance for the SNPs showing the most significant associations. RegulomeDB showed a score of 4 suggesting the presence of a transcription factor binding motif and a DNase sensitivity peak for rs2518719. HaploReg also suggested the presence of a DNase sensitivity peak and the polymorphism to alter the sequence recognized by the Neuron-Restrictive Silencer Factor (NRSF) regulatory repressor. No significant association between rs2518719 and expression of any gene is reported in the GTEx project. We used The SNAP software to find SNPs in LD with rs2518719 and we found 9 variants that had a minimum LD of 0.760 (rs2188127, rs3731222, rs3731217, rs3731204, rs3731198, rs2811711, rs495490, rs575427, rs647188) but also for them there was no evidence of association with gene expression in GTEx.

## Discussion

The background of common genetic susceptibility to sporadic PNETs is largely unknown. The rarity of the disease is certainly one motivation for the scarcity of the information on the disease genetic susceptibility. Only a small number of studies have been performed with the largest study having 101 cases and 432 controls^11^. To further our understanding on the topic we conducted the largest study on the disease, with up to 320 cases and 4,436 controls, taking advantage of the mainframe of the Pancreatic Disease Research (PANDoRA) Consortium. The only finding of potential significance was that the carriers of the rare A allele of the rs2518719 SNP had an increased risk of developing the disease. This SNP belongs to the *CDKN2A* gene and lies in the second intron of the gene around 2000 bp from the start of the 3′UTR. Rs2518719 is in tight LD with another variant in the gene, rs3731217 (r^2^ = 0.925, D’ = 1 in Caucasian, as reported by 1000 Genomes), that lies in the first intron of the gene. This latter SNP is a well known pleiotropic susceptibility polymorphism and it has been found to be associated with increased risk of developing childhood acute lymphoblastic leukemia^28^,^29^, differentiated thyroid carcinoma^30^ and salivary gland carcinoma^31^. In addition the SNP was also reported to modulate the survival of oropharingeal cancer patients^32^. Despite all these evidences, indicating a role of the variant allele in developing various diseases, no functional studies have been performed and therefore it is not possible to elucidate a possible direct effect of the SNP. It has been suggested that rs3731217 might be involved in the regulation of the p53 gene expression, but given the before mentioned lack of direct functional evidence this remains highly speculative^32^.

The observation that it is always the same allele, in different cancer types, to be associated with an increased risk indicates a pleiotropic role for rs2518719/rs3731217 (or one of the other variants in tight LD) and also strongly suggests that the causal variant alters the function of the protein, the regulation of the gene expression or both in a way that influences the chances of developing cancer in different organs. The 9p21.3 locus in general, and the *CDKN2A/CDKN2B* genes in specific, are a classic examples of pleiotropic regions since they are associated with a very large number of human traits and diseases^17^,^18^,^19^,^22^,^23^,^24^. Pleiotropic regions are probably more accessible DNA stretches than normal and therefore variability within them may result to be non neutral more likely than in any other randomly selected DNA sequence. However the regulation of pleiotropic region is likely to be more complex than other genome parts and therefore this increases the difficulties in understanding the effect of the genetic effect at each single locus.

The results from HaploReg suggested that rs2518719 could alter the sequence recognized by the NRSF regulatory repressor. NRSF is encoded by the RE1-Silencing Transcription factor (*REST*) gene, and its deregulation has been associated to the development of several tumors including colon and lung cancer^33^. Therefore a possible explanation of the pleiotropic valence of this SNP may rely on the regulatory effect of the sequence recognized by NRSF/REST. However, even if it is intriguing, also this functional explanation remains speculative especially considered that NRSF is primarily involved in the silencing of neural genes in non neuronal tissues^34^. The lack of reliable functional data and eQTLs for the SNP can be explained by the fact that the entire *CDKN2A/2B* region is under a very complex gene regulation.

The results from Ter-Minassian and colleagues, in their study on neuroendocrine tumors, are in agreement with what we found suggesting, also in their case, an increased risk specifically of PNET for individuals carrying the rare allele for rs2518719 and the linked rs3731217 and rs3731198^11^. In that paper the authors also observed an independent signal from the rs3731211 variant. Considering our sample size and their ORs we had a power greater than 99% of finding the association but we did not. Considering also the p value reported (p = 0.042) the most likely explanation for this discrepancy is that the association was due to statistical fluctuation due to the rarity of the disease.

In the light of multiple testing this association is not statistically significant, however considering the concordance with previous reports and the low statistical power permitted by the rarity of the disease a Bonferroni correction is too strict. We therefore used also the False Positive Report Probability (FPRP^35^) and using a prior of 0.25 the association retains noteworthiness (posterior p = 0.188). We used a prior p = 0.25 based on the fact that the polymorphism is pleiotropic or in almost complete LD with one, and that it has been found to be associated already with PNET risk. We are aware that the results given by the FPRP are indications and that the final confirmation of the association can be only given by functional studies.

The present study carries some limitation, such as limited clinical information on the sporadic PNETs patients in terms of environmental and familial risk factors and disease stage and grade. However, our results, taken together with what found by Ter-Minassian and colleagues convincingly suggest that the pleiotropic *CDKN2A* region is associated with the risk of developing PNETs as already observed for several other cancer types.

## Materials and Methods

### Study population

In the present study 320 sporadic PNET patients and 4,436 controls belonging to the Pancreatic Disease Research (PANDoRA) consortium were recruited in 4 European countries. Cases were sporadic, i.e. not observed in the context of genetic syndromes associated with PNET, such as MEN-1, MEN-2, VHL or TSC. Controls were recruited in the same hospitals, or at least geographical region from where the cases were recruited. All participants signed a written consent form. The study protocol was approved by the ethic board of the University of Heidelberg and was carried out according to declaration of Helsinki. Additional information on the PANDoRA consortium have been given elsewhere^36^.

### SNPs selection

We investigated the common genetic variability in the *CDKN2A*/*B* region using tagging SNPs and potentially functional SNPs. Tagging SNPs were selected using a pairwise tagging method with a minimum r^2^ of 0.8 and a minor frequency allele of 0.05. We identified 11 SNPs that efficiently tagged the selected gene region. Subsequently we added two (rs1063192 and rs3217992) putative miR-SNPs that are polymorphic variants predicted to alter the binding of one or more microRNAs to their target. Therefore the final selection resulted in 13 SNPs.

### Sample preparation and genotyping

For each sample DNA was extracted from whole blood using the AllPrep Isolation Kit (Qiagen, Hilden, Germany) or the Qiagen-mini kit (Qiagen, Hilden, Germany), according to the manufacturer’s protocol. Blood was kept frozen before the extraction. Genotyping was performed using KASP (KBioscence, Hoddesdon, UK) and TaqMan (Thermo Fisher Scientific, Waltham, MA, USA) technologies. Genotyping was carried out using 384 well plates using 5ng of DNA for each sample. The order of DNA samples was randomized on plates in order to ensure that similar numbers of cases and controls were analyzed in each batch. Detection was performed using an ABI PRISM Viia7 sequence detection system with Viia7 software (Applied Biosystems, Foster City, CA, USA). The personnel performing the genotyping was blinded on the identity of the subject (i.e. whether the DNA belonged to a case or a control subject). For quality control, duplicates of 10% of the samples were interspersed throughout the plates. In addition, we discarded all the samples that had a call rate < 75%.

### Statistical analysis

Using Pearson’s chi-square test we checked the departure from Hardy–Weinberg equilibrium (HWE) for all SNPs in the control subjects of the study. Unconditional logistic regression computing odds ratios (OR), 95% confidence intervals (95% CIs) and p values was used to estimate the association between the genotypes of all polymorphisms and PNET risk. The more common allele among the controls was assigned as the reference category and the co-dominant model inheritance model was assessed. All analyses were adjusted for age, gender and geographic origin.

### Multiple testing

We used two methods to correct for multiple testing: a robust conservative test and a Bayesian one. The threshold to declare an association to be significant with a Bonferroni correction is 0.0038 (0.05/13). Considering the vast *a priori* knowledge on the region and on the SNPs in particular we opted to use also the False Positive Report Probability (FPRP) method. The FPRP was developed by Wacholder and colleagues^35^ to assess if an association is ‘noteworthy’ using a Bayesian approach that includes a priori knowledge of the variable taken in consideration. For associations with moderate to high prior evidence (e.g. association reported in a previous study, convincing functional evidence) the prior probabilities used are in the range 0.10–0.25, whereas lower prior probabilities are employed with decreasing information on the SNP and/or the relation between the SNP and the disease^35^,^37^.

### Bioinformatic analysis

We used several bioinformatic tools to assess the possible functional relevance for the SNP showing the most significant association with risk of developing PNET. RegulomeDB ( http://regulome.stanford.edu/)^38^ and HaploReg v2B^39^ were used to identify the regulatory potential of the region nearby each SNP. The GTEx portal web site^40^ was used to identify potential associations between the SNP and expression levels of nearby genes (eQTL). In addition we used the SNAP software^41^ to find SNPs in LD with the SNP that showed the strongest association with PNET risk using a threshold of r^2^ = 0.70.

## Additional Information

**How to cite this article**: Campa, D. *et al*. Common germline variants within the *CDKN2A/2B* region affect risk of pancreatic neuroendocrine tumors. *Sci. Rep.* **6**, 39565; doi: 10.1038/srep39565 (2016).

**Publisher's note:** Springer Nature remains neutral with regard to jurisdictional claims in published maps and institutional affiliations.

## Figures and Tables

**Table 1:** Study population.

	Cases	Controls
Region		
Germany	39	2,282
Greece	23	175
Northern Italy	88	595
Central Italy	146	549
Southern Italy	11	500
Poland	13	335
Total	320	4,436
Sex		
Male	162	2,305
Female	156	2,071
**Median age**	58	58
**(interquartile range)**	(48–66)	(47–64)

**Table 2:** Associations between selected SNPs in the *CDKN2A/2B* region and PNET risk.

SNP	Pos chr 9 (Hg18)	Alleles (M/m)^a^	Cases^b^			Controls^b^			MM vs Mm			MM vs mm			MM vs Mm + mm		
			MM	Mm	mm	MM	Mm	mm	OR^c^	95% CI^c^	p	OR^c^	95% CI^c^	p	OR^c^	95% CI^c^	p
rs3731257	21,956,221	C/T	166	130	21	2,243	1,700	290	1.07	0.81–1.40	0.640	0.86	0.49–1.53	0.610	1.04	0.80–1.35	0.786
rs11515	21,958,199	C/G	235	83	3	3,065	1,114	108	1.03	0.77–1.39	0.834	0.28	0.07–1.14	0.076	0.95	0.71–1.28	0.758
**rs2518719**	21,960,427	G/A	228	78	10	3,217	996	93	1.07	0.78–1.47	0.671	**2.08**	**1.05–4.11**	**0.035**	1.16	0.86–1.56	0.336
rs3731249	21,960,916	C/T	295	27	0	3,484	310	9	1.06	0.66–1.70	0.815	N/A			1.16	0.63–1.63	0.949
rs3731246	21,961,989	C/G	237	61	2	1,630	450	29	0.89	0.62–1.26	0.501	0.57	0.14–2.43	0.451	0.87	0.61–1.22	0.412
rs2811708	21,963,422	T/G	160	126	27	2,200	1,633	304	1.14	0.86–1.51	0.354	1.20	0.73–1.97	0.463	1.15	0.88–1.50	0.299
rs3731239	21,964,218	C/T	132	147	36	1,851	1,909	534	1.06	0.80–1.40	0.700	0.84	0.53–1.33	0.455	1.01	0.77–1.32	0.944
rs3731211	21,976,847	A/T	144	132	30	1,844	1,457	340	1.21	0.91–1.61	0.191	1.15	0.715–1.83	0.572	1.20	0.91–1.57	0.193
rs2811710	21,981,923	T/C	127	150	42	1,086	1,336	433	0.97	0.73–1.30	0.842	0.96	0.64–1.44	0.835	0.97	0.74–1.27	0.814
rs3218009	21,988,757	C7G	270	51	1	3,541	728	50	1.01	0.71–1.43	0.965	0.38	0.05–2.82	0.346	0.96	0.68–1.37	0.862
rs3217992	21,993,223	G/A	98	163	57	1,456	2,068	723	1.07	0.80–1.44	0.649	1.04	0.70–1.54	0.862	1.06	0.80–1.41	0.675
rs1063192	21,993,367	G/A	119	139	50	1,436	1,809	549	0.95	0.71–1.28	0.738	1.27	0.86–1.87	0.227	1.02	0.78–1.35	0.865
rs3217986	21,995,330	C/A	283	35	3	3,672	671	24	0.68	0.44–1.03	0.066	2.45	0.72–8.35	0.153	0.73	0.49–1.09	0.124

^a^M: major allele; m: minor allele.

^b^Numbers may not add up to the total of available subjects due to genotyping failure.

^c^OR: odds ratio; CI: confidence interval. All analyses were adjusted by age, sex and geographical region of origin. Results in bold are statistically significant (p < 0.05).
